# Early Detection of Symptom Exacerbation in Patients With SARS-CoV-2 Infection Using the Fitbit Charge 3 (DEXTERITY): Pilot Evaluation

**DOI:** 10.2196/30819

**Published:** 2021-09-16

**Authors:** Kan Yamagami, Akihiro Nomura, Mitsuhiro Kometani, Masaya Shimojima, Kenji Sakata, Soichiro Usui, Kenji Furukawa, Masayuki Takamura, Masaki Okajima, Kazuyoshi Watanabe, Takashi Yoneda

**Affiliations:** 1 Department of Cardiovascular Medicine Graduate School of Medical Sciences Kanazawa University Kanazawa Japan; 2 Department of Health Promotion and Medicine of the Future Kanazawa University Kanazawa Japan; 3 Health Care Center Japan Advanced Institute of Science and Technology Ishikawa Japan; 4 Intensive Care Unit Kanazawa University Hospital Kanazawa Japan; 5 Japan Community Health Care Organization Kanazawa Hospital Kanazawa Japan

**Keywords:** COVID-19, silent hypoxia, wearable device, Fitbit, estimated oxygen variation, detection, infectious disease, pilot study, symptom, outpatient, oxygen, sleep, wearable

## Abstract

**Background:**

Some patients with COVID-19 experienced sudden death due to rapid symptom deterioration. Thus, it is important to predict COVID-19 symptom exacerbation at an early stage prior to increasing severity in patients. Patients with COVID-19 could experience a unique “silent hypoxia” at an early stage of the infection when they are apparently asymptomatic, but with rather low SpO_2_ (oxygen saturation) levels. In order to continuously monitor SpO_2_ in daily life, a high-performance wearable device, such as the Apple Watch or Fitbit, has become commercially available to monitor several biometric data including steps, resting heart rate (RHR), physical activity, sleep quality, and estimated oxygen variation (EOV).

**Objective:**

This study aimed to test whether EOV measured by the wearable device Fitbit can predict COVID-19 symptom exacerbation.

**Methods:**

We recruited patients with COVID-19 from August to November 2020. Patients were asked to wear the Fitbit for 30 days, and biometric data including EOV and RHR were extracted. EOV is a relative physiological measure that reflects users’ SpO_2_ levels during sleep. We defined a high EOV signal as a patient’s oxygen level exhibiting a significant dip and recovery within the index period, and a high RHR signal as daily RHR exceeding 5 beats per day compared with the minimum RHR of each patient in the study period. We defined successful prediction as the appearance of those signals within 2 days before the onset of the primary outcome. The primary outcome was the composite of deaths of all causes, use of extracorporeal membrane oxygenation, use of mechanical ventilation, oxygenation, and exacerbation of COVID-19 symptoms, irrespective of readmission. We also assessed each outcome individually as secondary outcomes. We made weekly phone calls to discharged patients to check on their symptoms.

**Results:**

We enrolled 23 patients with COVID-19 diagnosed by a positive SARS-CoV-2 polymerase chain reaction test. The patients had a mean age of 50.9 (SD 20) years, and 70% (n=16) were female. Each patient wore the Fitbit for 30 days. COVID-19 symptom exacerbation occurred in 6 (26%) patients. We were successful in predicting exacerbation using EOV signals in 4 out of 5 cases (sensitivity=80%, specificity=90%), whereas the sensitivity and specificity of high RHR signals were 50% and 80%, respectively, both lower than those of high EOV signals. Coincidental obstructive sleep apnea syndrome confirmed by polysomnography was detected in 1 patient via consistently high EOV signals.

**Conclusions:**

This pilot study successfully detected early COVID-19 symptom exacerbation by measuring EOV, which may help to identify the early signs of COVID-19 exacerbation.

**Trial Registration:**

University Hospital Medical Information Network Clinical Trials Registry UMIN000041421; https://upload.umin.ac.jp/cgi-open-bin/ctr_e/ctr_view.cgi?recptno=R000047290

## Introduction

The COVID-19 pandemic caused by SARS-CoV-2 has resulted in over 168 million cases and 3.5 million deaths worldwide as of late May 2021 [1]. The virus, with a long latency period of 2 to 14 days from initial infection to the onset of symptoms, is most transmissible just before symptoms appear [2]. Approximately half of infected patients are asymptomatic in some areas [3] but can spread the infection to others. Therefore, being asymptomatic might be one of the leading causes behind the global spread of the disease [4].

Some patients with COVID-19 experience rapid deterioration after 1 week of initial symptom onset, requiring oxygen or care in the intensive care unit (ventilators and extracorporeal membrane oxygenation [ECMO]) for severe pneumonia and acute respiratory distress syndrome–like symptoms [5]. According to a New York City report, the mortality rate of COVID-19 for patients on ventilators is over 75% [6]. The benefit of antiviral medication such as remdesivir or dexamethasone might be most apparent when it is used before symptom exacerbation [7,8]. Thus, it is important to predict COVID-19 symptom exacerbation at an early stage before patients experience increasing severity. In Japan, an increasing number of patients with COVID-19 with mild symptoms are managed in their homes or hotels to make effective use of medical resources [9]. However, in some cases, worsening symptoms resulted in death before the patient could be admitted to a hospital [10,11]. Therefore, it is necessary to construct an alarm system to detect signs of severity beforehand to prevent patients from serious illness or death while waiting at home or in hotels.

To evaluate the severity of pulmonary diseases, blood oxygen saturation (SpO_2_) levels, measured by pulse oximetry, usually provides important information [12]. In fact, patients with COVID-19 could experience a unique “silent hypoxia” at an early stage of the infection when they are apparently asymptomatic, but have rather low SpO_2_ levels [13]. Since a low SpO_2_ normally indicates a severe pulmonary reaction to the disease, monitoring SpO_2_ could provide potential biometric data to predict impending disease deterioration [14]. Indeed, low SpO_2_ levels could predict future disease exacerbation in patients with chronic obstructive pulmonary disease [12]. However, except in special situations in an intensive care unit, continuous SpO_2_ monitoring in daily life is unlikely because the measuring equipment normally needs to be clipped to one’s finger for every measurement.

Recently, high-performance wearable devices, such as the Apple Watch and Fitbit, have become commercially available to monitor biometric data including steps, resting heart rate (RHR), physical activity, sleep quality, and even estimated oxygen variations (EOV; a relative physiological measure of a user’s SpO_2_ levels during sleep). For example, one study used biometric data obtained from a Fitbit, a smartwatch device worn on one’s wrist, and showed that increased RHR and decreased sleep duration were associated with flu-like symptoms [15]. With COVID-19, some studies have demonstrated that changes in certain biometric indicators, including RHR, sleep duration, or respiratory rate, from the baseline might predict the occurrence of COVID-19 symptoms before their onset among those who use wearable devices on a daily basis [16-18]. However, it is still unclear whether the detection of variations in blood oxygen level might be useful for predicting COVID-19 severity by wearable devices, or whether wearable devices can detect signs of symptom exacerbation in patients with a confirmed case of COVID-19.

Here, we conducted the DEXTERITY pilot study, leveraging a wearable device to obtain biometric data, EOV and RHR, in particular, in patients diagnosed with COVID-19 to predict symptom exacerbation.

## Methods

### Participants

We prospectively recruited 28 patients with a positive SARS-CoV-2 polymerase chain reaction (PCR) test, according to the study protocol approved by the Kanazawa University and the Japan Community Health Care Organization (JCHO) Kanazawa Hospital Institutional Review Boards. This study was conducted from August to November 2020 at JCHO Kanazawa Hospital in Kanazawa, Japan. We performed the study in compliance with the Ethical Guidelines for Medical and Health Research Involving Human Subjects, the Declaration of Helsinki, and other guidelines in Japan. We registered this study with the University Medical Information Network Clinical Trial Registry on August 14, 2020 (UMIN000041421).

We included patients who were diagnosed with COVID-19 and had a positive SARS-CoV-2 PCR test result within 1 week before enrollment in the study. We excluded patients who met the following criteria: (1) unable to wear and use the wearable device, (2) unable to connect the wearable device to the smartphone app, (3) unable to download or use the smartphone app, and (4) unable to provide informed consent because of severe COVID-19 symptoms. We obtained informed consent electronically via mobile platforms using the Research Electronic Data Capture (REDCap) system from all participants.

### Wearable Device and Data Extraction

We provided a Fitbit Charge 3 to each participant. They wore the wearable device for 30 days to detect COVID-19 symptom exacerbation, which normally occurs at 7 to 14 days after the onset of initial symptoms [5]. The Fitbit Charge 3 was connected to each patient’s smartphone via the Fitbit app, and their biometric data, including RHR and sleep quality, were extracted through the Fitabase, a web-based Fitbit-derived data extraction system for clinical studies.

Patients were asked to complete electronic questionnaires in the REDCap system, which included questions on baseline characteristics (age; sex; height; weight; BMI; smoking status [current, former, or never]; presence of hypertension, diabetes mellitus, or dyslipidemia; any other medical history, and any medication use) at the time of enrollment. Additionally, we obtained information regarding COVID-19–related symptoms from the patients during hospitalization and after discharge over the course of the 30-day study period. COVID-19–related symptoms included fever, cough, fatigue, difficulty in breathing, nausea, diarrhea, dysosmia, or dysgeusia. We also conducted weekly checks via telephone on symptom improvement or exacerbation, readmission to the hospital, oxygenation, use of mechanical ventilation, use of ECMO, or death resulting from all causes.

### Estimated Oxygen Variation

**Figure 1:**
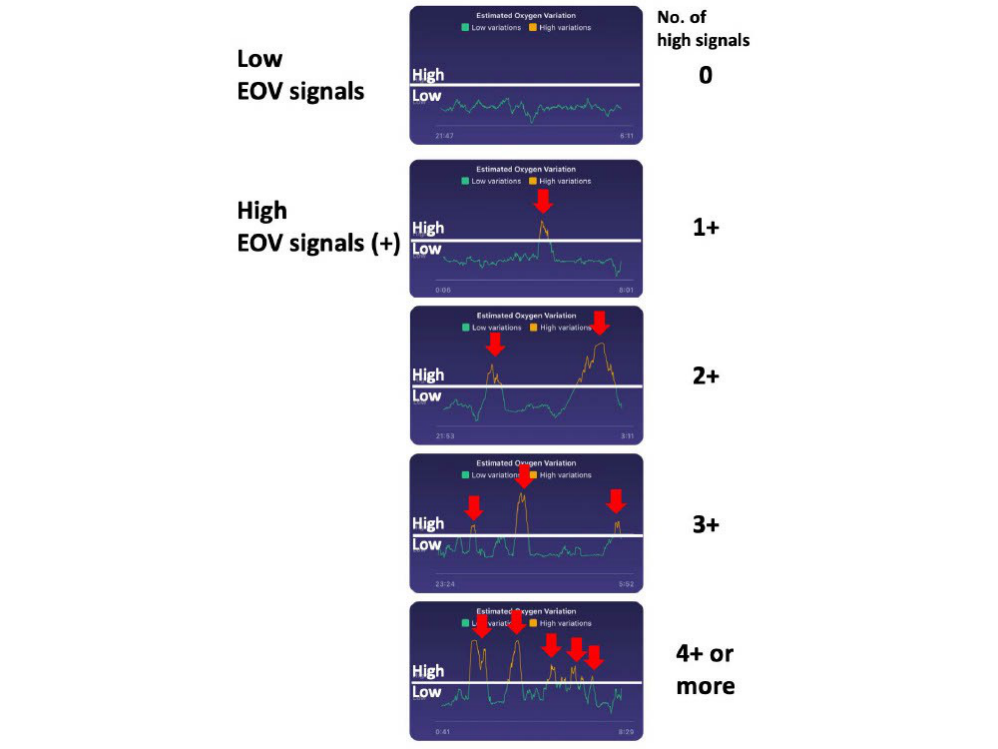
Representative graphs of low (negative) and high (positive) estimated oxygen variation (EOV) signals from the Fitbit Charge 3.

In addition to the biometric data obtained from the Fitbit Charge 3, we directly collected daily EOV graphs by taking screenshots of each patient’s app interface. EOV is internally calculated using an algorithm that estimates the variation in the reflected rate from the reflected optical signals every minute. If a patient’s oxygen level is stable, the variation is low or close to zero. However, if a patient’s oxygen level exhibits a significant dip and recovery within the index period, the variation shows a high signal. Variations that cross the threshold line are shown in Figure 1. We defined a high EOV (single day) signal as an EOV value that passes the threshold line on the graph at one or more times during sleep. Since we considered that a symptom deterioration signal could last several days, if a high single-day EOV signal continues for 2 or more consecutive days, we regarded the signal as a high EOV signal and calculated the sensitivity, specificity, positive predictive value (PPV), and negative predictive value (NPV).

### Outcomes

The primary outcome was the composite of deaths by all causes, use of ECMO, use of mechanical ventilation, oxygenation, and exacerbation of COVID-19 symptoms, irrespective of readmission. We also assessed each outcome individually as secondary outcomes. We made phone calls to each patient every week and asked whether their symptoms were stable or had changed. If the patient’s symptoms changed, we ask when it occurred, and 2 study investigators judged if the change was deemed to be an exacerbation or not. We defined exacerbation of COVID-19 as fever, dyspnea or intense malaise, and other common cold symptoms such as cough, gastrointestinal symptoms, or symptoms considered similar to those seen when the patient had COVID-19. If the patient did not answer the call, we contacted the patient's family instead and instructed them to ask the patient to pick up the phone. If that did not work, we called on a different day of the week.

### Statistical Analysis

The baseline profile is shown as the mean (SD) or median with quantiles (for continuous variables), or proportions (for categorical variables). We overlayed and compared the onset of the outcomes and the number of days high EOV signals were detected. We defined a successful prediction of an outcome by EOV as the presence of high EOV signal(s) within 2 days of the onset of the outcome. We also defined high RHR signals as a daily RHR exceeding 5 beats/day compared with the minimum RHR of each patient during the study period. We calculated the sensitivity, specificity, PPV, and NPV for both the high EOV signal and the high RHR signal for primary outcome prediction. Sensitivity was defined as the number of true positives divided by the number of symptom exacerbation events, where a true-positive event refers to the appearance of a high EOV signal and exacerbated symptoms. Specificity was defined as the number of true negatives divided by the number of events without exacerbated symptoms, where a true negative refers to an event where a high EOV signal does not appear and the patient’s symptom does not exacerbate. PPV was defined as the number of true positives divided by the number of high EOV signals. NPV was defined as the number of true negatives divided by the number of EOV signals that are not high. All tests were two-sided, and significant differences were considered when *P*<.05. We used R, version 3.6.1 (R Foundation for Statistical Computing) for the analyses.

### Data and Code Availability

The anonymized data set of this study will be available from the corresponding author upon publication. The investigator may only use the data for the purpose outlined in the request. Data redistribution is prohibited.

Custom codes or mathematical algorithms were not used in this study.

## Results

### Study Participants and Baseline Characteristics

**Figure 2:**
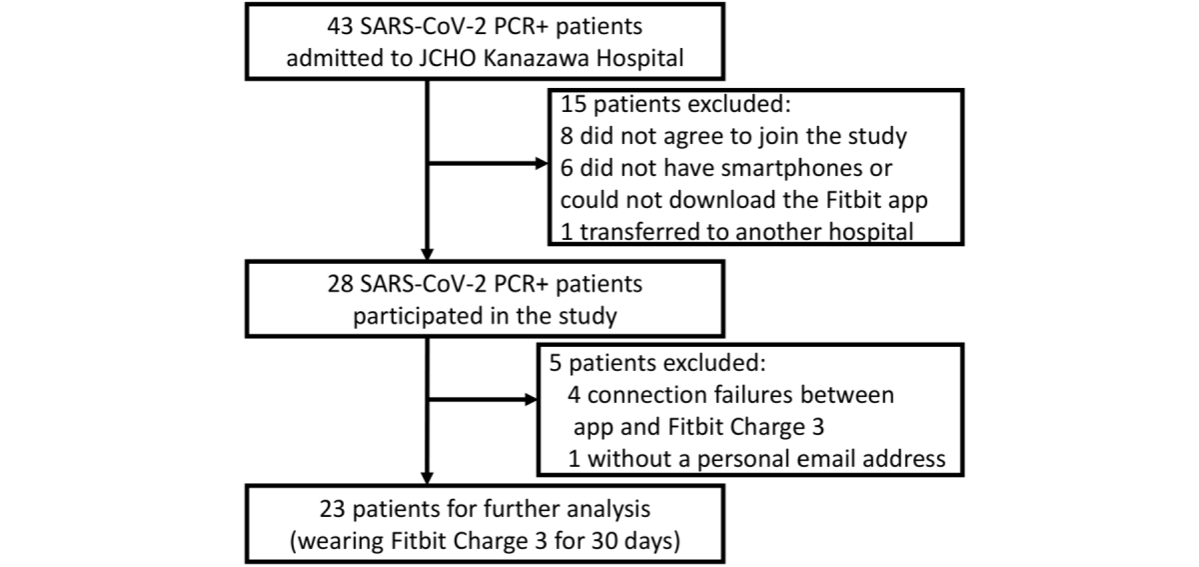
Study flowchart. JCHO: Japan Community Health Care Organization; PCR: polymerase chain reaction.

During the study period, 43 patients were admitted to JCHO Kanazawa Hospital. Of these, 15 were excluded from the study; 8 did not consent to join the study; 6 did not have smartphones or could not download the Fitbit app; and 1 had a severe respiratory condition and was immediately transferred to another hospital. Thus, we prospectively recruited 28 SARS-CoV-2 PCR-positive patients in this pilot study. Of this sample, 4 patients could not connect to the Fitbit account, and 1 patient had no personal email address. Therefore, 23 patients were followed up for 30 days and included in further analyses (Figure 2).

Table 1 shows the baseline characteristics of the patients. A total of 23 patients were included. The patients had a mean age of 50.9 (SD 20) years, and 70% (n=16) were female. Two (9%) patients had a history of malignancy (2 patients with breast cancer), but none had cardiovascular or cerebrovascular diseases. Symptoms at admission were dyspnea in 2 (9%) patients, fever in 12 (52%) patients, dysgeusia in 3 (13%) patients, dysosmia in 1 (4%) patient, sore throat in 1 (4%) patient, and no symptoms in 4 (17%) patients. The median interval from initial COVID-19 symptoms to Fitbit use was 5 days (range 1-9 days), and the median days of wearing the Fitbit was 19 (IQR 15.5-28) days.

**Table 1:** Baseline characteristics.

Characteristic		Participants (N=23)
Age (years), mean (SD)		50.9 (20)
Gender (female), n (%)		16 (70)
Body weight (kg), mean (SD)		58.7 (16)
BMI (kg/m^2^), mean (SD)		22.8 (4.7)
**Comorbidities**		
	Hypertension, n (%)	5 (22)
	Diabetes mellitus, n (%)	2 (9)
	Dyslipidemia, n (%)	5 (22)
**Medical history, n (%)**		
	Malignancy	2 (9)
	Cardio- or cerebrovascular diseases	0 (0)
**Smoking status, n (%)**		
	Never	12 (52)
	Former	9 (39)
	Current	2 (9)
**Symptoms at admission, n (%)**		
	Fever	12 (52)
	Dysgeusia	3 (13)
	Dyspnea	2 (9)
	Dysosmia	1 (4)
	Sore throat	1 (4)

### Estimated Oxygen Variation and Outcomes

**Figure 3:**
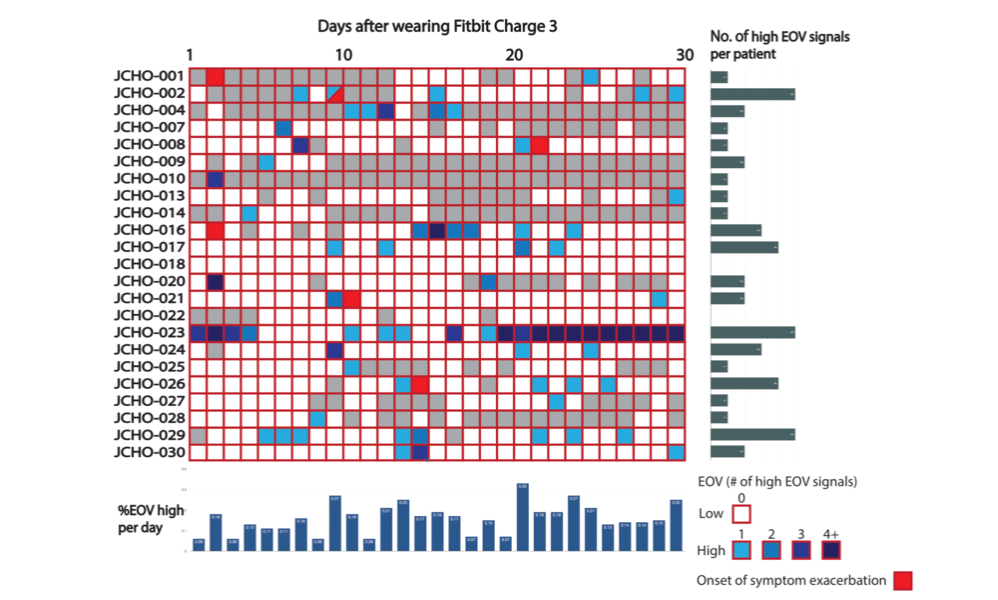
Summary of high estimated oxygen variation (EOV) signals and events among patients with a positive SARS-CoV-2 polymerase chain reaction test. The vertical columns represent the ID of each patient, and the horizontal axis represents the number of days of wearing a Fitbit. Blue squares represent a single-day high EOV signal. Gray columns denote the days when no EOV data were obtained. Red columns indicate the days that primary outcomes (symptom exacerbation events) occurred. JCHO: Japan Community Health Care Organization.

Figure 3 demonstrates a summary of high EOV signals among the 23 patients. We observed 48 high EOV signals (73 single-day high signals) during the study. We found a median of 1 high EOV signal per patient (IQR 1-3). The median percentage of EOV per day was 16% (IQR 11%-19%). Of the 23 patients, we excluded 1 patient (JCHO-023) from further analyses because of obstructive sleep apnea syndrome (OSAS) detected via a polysomnography (the details are described in the “Representative Case 3: Obstructive Sleep Apnea Syndrome (JCHO-023)” section).

The primary outcomes (symptom exacerbation events) occurred 7 times in 6 patients during the study period. The primary outcomes included the use of high-flow nasal cannula (HFNC) (n=2), exacerbation of cough (n=2; 1 patient was readmitted to the hospital), exacerbation of dysosmia (n=1), and experience of fever and general malaise (n=1). In patients with EOV data, we successfully observed high EOV signals within 2 days of the symptom exacerbation events in 4 out of 5 cases (sensitivity=80%, specificity=90%), although NPV was 99.7% and PPV was only 9.3%. The reference sensitivity, specificity, PPV, and NPV of high RHR signals for detecting these events were 50%, 88%, 6.7%, and 99.1%, respectively, all of which were lower than those of high EOV signals. The clinical course of the patients with COVID-19 are shown in Multimedia Appendix 1.

**Figure 4:**
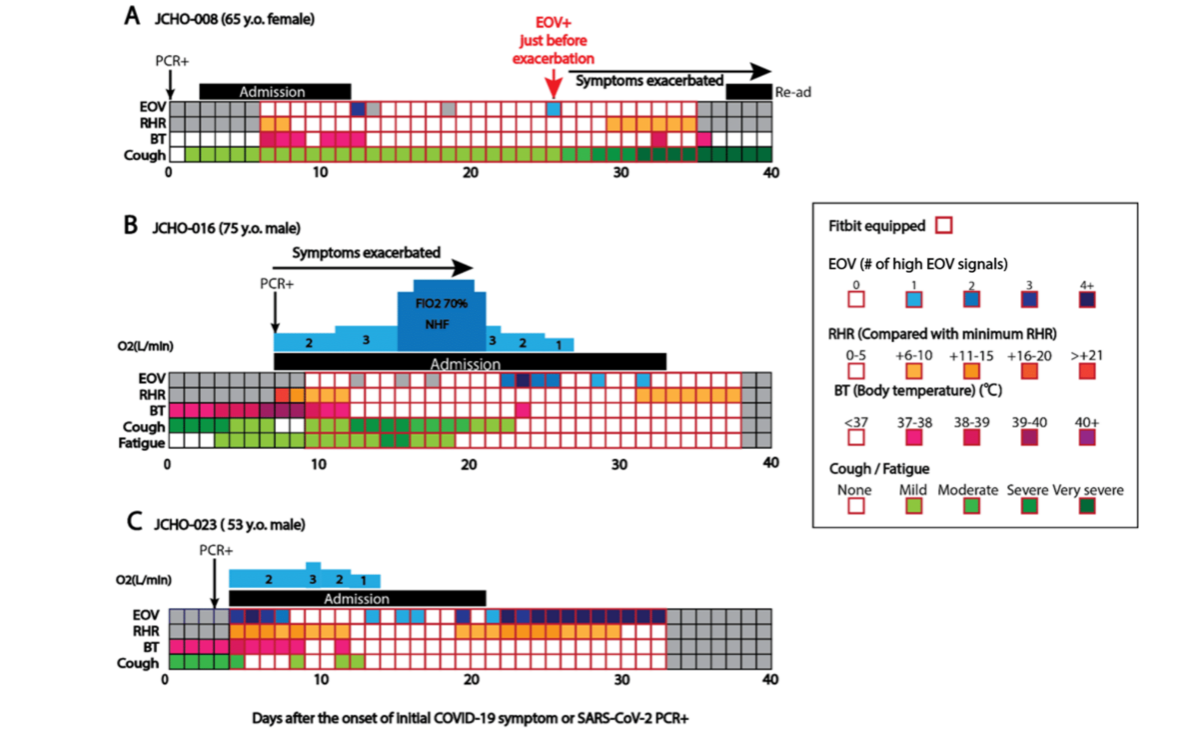
The clinical courses of patients JCHO-008, JCHO-016, and JCHO-023. The vertical columns represent estimated oxygen variation (EOV), resting heart rate (RHR) (compared with minimum RHR), body temperature (BT), and cough or fatigue severity levels. The horizontal axis represents the number of days after a positive SARS-CoV-2 polymerase chain reaction (PCR) test (JCHO-008) or the occurrence of COVID-19–related symptoms (JCHO-016 and JCHO-023). Gray columns represent a day when no EOV data were obtained. FiO_2_: fraction of inspired oxygen; JCHO: Japan Community Health Care Organization; NHF: nasal high flow; Re-ad: readmission.

Next, we reported representative cases in whom we could successfully observe (JCHO-008) or not observe (JCHO-016) high EOV signals just before the exacerbation of COVID-19 symptoms (Figure 4). In addition, we presented a patient with COVID-19 (JCHO-023) in whom we unintentionally detected OSAS due to the extreme and consistently high EOV signals observed during the study period.

### Representative Case 1: Successfully Detected (JCHO-008)

A 65-year-old woman with a history of gastric cancer was referred to our hospital due to a positive SARS-CoV-2 PCR test result (Figure 4A). She was asymptomatic at the time of diagnosis by PCR. However, she began coughing just before hospital admission, and her SpO_2_ was 98% at room air. Although she experienced a moderate fever (up to 38.8℃) with computed tomography (CT)–confirmed pneumonia for 1 week, she became afebrile and asymptomatic except for a slight cough on the day of discharge on day 12. A high EOV signal was once detected shortly after discharge, but no symptom exacerbation occurred after the signal. However, another high EOV signal occurred on day 25. Since her cough suddenly worsened following the fever after the signal, she visited a hospital on day 30. Her chest CT exhibited COVID-19–like infiltration and interstitial shadow, and she was readmitted to the hospital and diagnosed with COVID-19–induced pneumonia recurrence. In this case, the high EOV signal on day 25 was successfully observed just before the symptom exacerbation on day 26.

### Representative Case 2: Undetected (JCHO-016)

There was an undetected case of EOV for COVID-19 symptom exacerbation before symptom onset. A 75-year-old man with a history of hypertension was referred to our hospital due to a positive SARS-CoV-2 PCR test (Figure 4B). At admission, his body temperature was 39.6℃, he had an SpO_2_ of 98% (via O_2_ nasal canula at a rate of 2 L/min), and his chest CT showed no shadow compatible with COVID-19. However, his SpO_2_ gradually worsened, and he had to use HFNC to maintain oxygenation on day 9 (the maximum fraction of inspired oxygen [FiO_2_] was 70%). Chest CT on day 8 revealed ground-glass opacity compatible with COVID-19. During this exacerbation, no high EOV signals were observed. However, just after HFNC discontinuation on day 13, high EOV signals were observed daily from days 14 to 17, but no exacerbation of symptoms was found afterward. Chest CT on day 15 showed remarkable improvement of ground-glass opacity. He discontinued oxygenation on day 21 and was discharged from the hospital on day 28. After discharge, high EOV signals were detected twice, but again, no exacerbations of any symptoms were found after these signals.

### Representative Case 3: Obstructive Sleep Apnea Syndrome (JCHO-023)

There was a patient with COVID-19 in whom we coincidentally detected OSAS due to the consistently high EOV signals observed during the study period. A 53-year-old man with a history of hypertension was referred to our hospital due to a positive SARS-CoV-2 PCR test (Figure 4C). He experienced a mild fever (up to 37.8℃) and a moderate cough (4 days before admission). At admission, his body temperature was 38.4℃, his SpO_2_ level was 96% (O_2_ nasal canula, 2 L/min), and his chest CT showed shadows compatible with COVID-19. Just after admission, high EOV signals were observed from days 1 to 4, but no exacerbation of symptoms was found after these signals. His body temperature and cough improved gradually after admission, and he became afebrile and asymptomatic after day 9. His SpO_2_ also improved gradually, and he discontinued oxygenation on day 11. High EOV signals were observed on day 10, days 12 to 13, and day 16, but no symptom exacerbation occurred after these signals. He was discharged from the hospital on day 17 and was afebrile and asymptomatic thereafter. However, a long-lasting high EOV signal was observed from days 18 to 29 without any symptoms. At this time, we assumed that he might be having sleep apnea syndrome (SAS). He underwent polysomnography by a portable polysomnogram monitor (SAS-2200, Nihon Kohden). His 3% oxygen desaturation index was 33.6 per hour, indicating severe OSAS. In this case, the high EOV signal was observed not due to the exacerbation of COVID-19 symptoms but by the existing condition of OSAS.

## Discussion

### Principal Findings

This is the first prospective pilot study to assess whether EOV, a relative physiological measure that indicates continuous SpO_2_ variations during sleep, using a Fitbit wearable device, could predict early exacerbation signs of SARS-CoV-2 infection before their onset. We demonstrated that the high EOV signals observed just before symptom exacerbation in 4 out of 5 cases (80%) was higher than that in RHR signals. In addition, we detected a severe case of OSAS via the intermittently high EOV signals obtained from the Fitbit device.

This study yielded several important findings. First, the high EOV signal provided by the Fitbit demonstrated a favorable sensitivity (80%) and high NPV (99.7%) (both higher than those of RHR signals) for COVID-19 symptom exacerbations prior to their onset. The high sensitivity and NPV of the device and signal used in this study are of particular importance for screening and early diagnosis of COVID-19 exacerbations, which could accurately identify cases that warrant closer inpatient or outpatient monitoring. In some patients with COVID-19, silent hypoxia has been reported, with remarkably low SpO_2_ levels, while having minimal typical symptoms such as fever, cough, or fatigue [19]. Following silent hypoxia, patients experienced apparent symptoms [20,21]. The mechanism of silent hypoxia involves a combination of many factors, including the response of the respiratory centers and the effect of comorbidities (eg, diabetes mellitus) and older age on breathing control [13]. Additionally, the idiosyncratic action of the coronavirus on receptors involved in chemosensitivity to oxygen has been demonstrated before [13]. Indeed, angiotensin-converting enzyme 2, the cell receptor of SARS-CoV-2, is expressed in the carotid body, the site at which the chemoreceptors sense oxygen [22]. The development of a thrombi within the pulmonary vasculature may also be related to silent hypoxia [21]. In this study, since silent hypoxia–like abrupt SpO_2_ depletion without apparent symptoms occurred in some patients, the EOV could successfully predict the exacerbation of COVID-19 symptoms. Although RHR is inferior to EOV in terms of sensitivity, it is not data that should be discarded, and there is a possibility that the prediction accuracy can be further improved by creating an index comprising both EOV and RHR.

Second, although high EOV signals showed high sensitivity and NPV for detecting COVID-19 symptom exacerbation, the PPV of high EOV signals was only 9.3%. High EOV signals may be invoked not only by SpO_2_ exacerbation but also by other situations including alcohol intake, emotional stress events, or medications, as heart rate increases in such situations [16]. Recent studies that aimed to predict the onset of COVID-19 showed that RHR- and sleep duration–derived indices tended to yield more false-positives among patients who had been diagnosed with COVID-19 [16,18]. The same situation might also have occurred for EOV. We still need to take into account the multiple factors that could interfere with a high EOV signal’s ability to predict COVID-19 symptom exacerbation.

Third, we detected a case of severe OSAS in a patient by chance due to consistently high EOV signals. To our knowledge, this is the first study to report a clinical case of extremely high EOV signals obtained by the Fitbit to detect SAS. SAS is a common disorder that causes patients to temporarily stop or decrease their breathing repeatedly during sleep [23]. It is caused by a dynamic upper airway collapse and results in low SpO_2_ during the night [24]. We believe that these SpO_2_ depletion events were detected by the Fitbit as high EOV signals. At present, Fitbit Inc is applying to the United States Food and Drug Administration to include this EOV function in a medical device to diagnose SAS. Once approved, Fitbit may soon become an innovative device to diagnose SAS.

### Limitations

This study has several limitations. First, the Fitbit wearable device, including its functions (eg, EOV) and analysis algorithms, was not yet approved as a medical device at the time of this study. Second, we could not acquire sufficient baseline data for each patient regarding RHR, EOV, and other biometric data because the duration of the study period over which the patients wore the Fitbit was only 30 days. Baseline biometric data for each physiological metric are very important to distinguish abnormal signals from normal variations at the individual level. In this study, we set the minimum value of each biometric factor during the study as the baseline value, but this could have affected the evaluation of positive signals for each metric. Third, we could not assess sleep duration as one of the biometric markers for exacerbation detection. We attempted to investigate the association of each sleep duration per day with the exacerbation of COVID-19 symptoms, but had to abandon it due to a lack of baseline data and significant variations between the in-hospital and after-discharge periods. Fourth, elderly patients who could not use a smartphone daily did not participate in the study, although they were more likely to have exacerbated SARS-CoV-2 infection symptoms. This problem can be solved by having medical staff operate the older patients’ smartphones instead under adequate infection protection. This, however, is difficult to do in a busy hospital ward. Fifth, the number of people in whom the primary outcome occurred (ie, 5) is too small to be generalizable. However, this is a pilot study, and future studies with a larger sample size will be needed to validate our results.

### Conclusions

In conclusion, we demonstrated that EOV from the Fitbit wearable device could detect 80% of symptom exacerbations among patients with SARS-CoV-2 infection before their onset. Additionally, we coincidentally detected OSAS through consistently high EOV signals. In the future, we hope to integrate EOV and other physiological metrics such as RHR, respiratory rate, or sleep data to improve the prediction accuracy of COVID-19 symptom exacerbations in advance.

## Appendix

Multimedia Appendix 1 The clinical course of the patients with COVID-19.

## Abbreviations

CT: computed tomography
ECMO: extracorporeal membrane oxygenation
EOV: estimated oxygen variation
FiO_2_: fraction of inspired oxygen
HFNC: high-flow nasal cannula
JCHO: Japan Community Health Care Organization
NPV: negative predictive value
OSAS: obstructive sleep apnea syndrome
PCR: polymerase chain reaction
PPV: positive predictive value
REDCap: Research Electronic Data Capture
RHR: resting heart rate
SAS: sleep apnea syndrome
SpO_2_: oxygen saturation
