# Supplementary figures and images for: Early Detection of Symptom Exacerbation in Patients With SARS-CoV-2 Infection Using the Fitbit Charge 3 (DEXTERITY): Pilot Evaluation
Source: JMIR Form Res. 2021 Sep 16;5(9):e30819. doi: 10.2196/30819 (PMC8448084; doi:10.2196/30819)

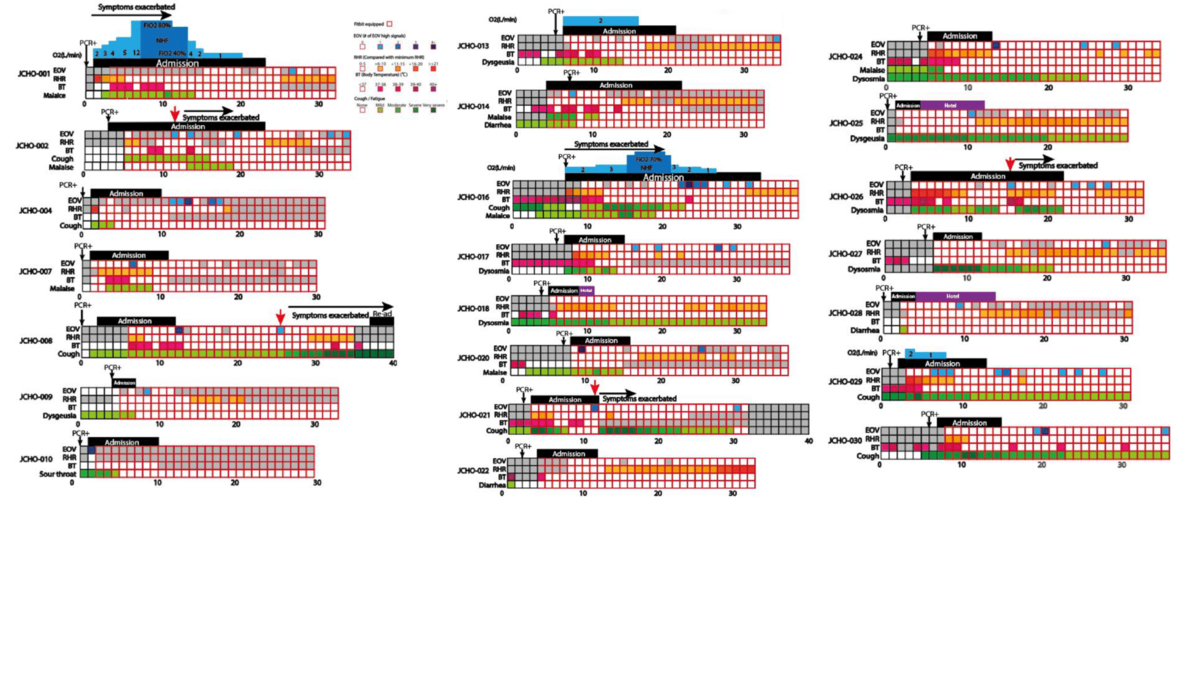

Supplement: Multimedia Appendix 1 [file formative_v5i9e30819_app1.png]
